# Fecal microbiota characteristics of Chinese patients with primary IgA nephropathy: a cross-sectional study

**DOI:** 10.1186/s12882-020-01741-9

**Published:** 2020-03-13

**Authors:** Xiaofang Hu, Jie Du, Yuhong Xie, Qiong Huang, Yi Xiao, Juan Chen, Siyuan Yan, Zhicheng Gong, Shaxi Ouyang

**Affiliations:** 1 Department of Pharmacy, Xiangya Hospital, Central South University, No. 87 Xiangya Road, Kaifu District, Changsha, 410008 Hunan China; 2 National Clinical Research Center for Geriatric Disorders, Xiangya Hospital, Central South University, No. 87 Xiangya Road, Kaifu District, Changsha, 410008 Hunan China; 3 Department of Nephrology, Hunan Provincial People’s Hospital, The first-affiliated hospital of Hunan normal university, No. 61 Jie-fang West Road, Fu-Rong District, Changsha, 410005 Hunan China

**Keywords:** Immunoglobulin A nephropathy, Fecal microbiota, Bacterial diversity, Gut microbiota dysbiosis

## Abstract

**Background:**

Growing evidence has shown that the gut-renal connection and gut microbiota dysbiosis play a critical role in immunoglobulin A nephropathy (IgAN). However, the fecal microbiome profile in Chinese patients with IgAN remains unknown. A cross-sectional study was designed for the first time to investigate the fecal microbiota compositions in patients with primary IgAN in China and to evaluate the relationship between the fecal microbiome and IgAN clinical presentation.

**Methods:**

Fecal samples were collected from 17 IgAN patients and 18 age-, sex-, and body mass index-matched healthy controls, and bacterial DNA was extracted for 16S ribosomal RNA gene sequencing targeting the V3-V4 region.

**Results:**

Fecal samples from the IgAN patients and healthy controls showed differences in gut microbiota community richness and compositions. Compared to the healthy controls, IgAN patients at the phylum level had an increased abundance of *Fusobacteria*, but a decreased abundance of *Synergistetes*. The significantly increased genera in the IgAN group were *Escherichia-Shigella, Hungatella,* and *Eggerthella,* all of which possess pathogenic potential. Furthermore, the genus *Escherichia-Shigella* was negatively associated with the estimated glomerular filtration rate (eGFR) but was positively associated with the urinary albumin-to-creatinine ratio (uACR). However, the genus *rectale_group* was present in the IgAN group with a low abundance and was negatively associated with the uACR. Functional analysis disclosed that infection-related pathways were enriched in the IgAN group.

**Conclusions:**

We demonstrate that gut microbiota dysbiosis occurs in patients with IgAN, and that changes in gut bacterial populations are closely related to IgAN clinical features, suggesting that certain specific gut microbiota may be a potential therapeutic target for IgAN.

## Background

Currently, immunoglobulin A nephropathy (IgAN) is the most common type of primary glomerular disease in adults worldwide [1, 2], accounting for approximately 58.2% of primary glomerular diseases in China [3]. The diagnosis of IgAN depends predominately on renal biopsy [4] to detect the deposition of glomerular mesangial immune complexes, which lead to inflammation and result in kidney failure [5]. IgAN has a wide spectrum of clinical features. While some IgAN patients develop the disease rapidly without noticeable symptoms [6], approximately 30–40% of the cases progress to end-stage renal disease (ESRD) after 20 to 30 years [7]. Although the overproduction of aberrantly glycosylated IgA1 [1] is regarded as an indispensable pathogenic contributor in IgAN, the exact pathogenic mechanism of IgAN is still poorly understood. As the dominant antibody isotype found in mucosal secretions, IgA plays a crucial role in controlling the composition of the gut microbiota by promoting symbiosis among bacteria [8], and secretory IgA has evolved to maintain diverse but stable gut microbial communities [9]. Mucosal abnormalities may be involved in the production of galactose-deficient IgA1, macromolecular immune complexes, and mesangial deposits [10]. Nevertheless, growing evidence has shown that the gut-renal connection and gut microbiota dysbiosis play a critical role in IgAN.

A gut–kidney axis has been implicated in causing chronic kidney diseases, in which a destroyed intestinal mucosal barrier facilitates bacterial lipopolysaccharide (LPS) entry into the circulation, resulting in uremic toxicity and systemic inflammation [11]. In regard to the importance of the gut-renal connection in IgAN [10], a subpopulation of γδ T cells that represent the majority of γδ T cells in normal gut mucosa has shown a significant decrease in the intestine of patients with IgAN [12]. Additionally, IgAN patients have high levels of serum B-cell activating factor (BAFF) and a proliferation-inducing ligand (APRIL), both of which are associated with the maintenance of tolerogenic immune responses to the microbiota [13, 14]. BAFF-overexpressing transgenic mice have mesangial deposits of IgA along with high circulating levels of aberrantly glycosylated polymeric IgA, which are associated with IgA-related renal diseases [13]. A recent genome-wide association study has revealed that most IgAN-related loci are associated with immune-mediated inflammatory bowel disease, intestinal barrier maintenance, and response to gut pathogens [15]. Collectively, cumulative evidence indicates that the disorder of intestinal mucosal immune response is the culprit behind the development of IgAN. However, intestinal microbes growing on the mucosal surface are in contact with intestinal epithelial cells and play a regulatory role in the intestinal immune system by altering intestinal permeability and interacting with Toll-like receptors (TLRs) expressed on the mucosal surface cells [16]. Thus, the commensal microbes interact with and influence the host’s innate and adaptive immune systems. For example, mouse segmented filamentous bacteria promote the accumulation of proinflammatory T helper 1 (Th1) and Th17 cells in the gut-associated lymphoid tissues [17], whereas other microbes, such as *Bacteroides fragilis* [18] and *Faecalibacterium prausnitzii* [18], enhance the accrual of T regulatory cells. Additionally, Grosserichter-Wagener et al. have reported that the composition of intestinal bacteria can affect the antibody reaction activity, Th cell subpopulation, and IgA reaction activity [19]. In recent years, fecal microbiota transplantation (FMT) has been proved to effectively rebuild the intestinal microecological balance. A prospective cohort trial to evaluate the safety and efficacy of FMT in IgAN patients is ongoing [20]. Hence, the gut microbiota is critical to maintaining intestinal immune homeostasis, and defective mucosal microenvironments and unbalanced gut microbiota might be important contributing factors toward the pathogenesis of IgAN [10].

The intestinal microbiota dysbiosis associated with IgAN has previously been studied in Italy [21]. However, geographical origin and dietary habits have a greater impact on gut microbial communities than body mass index (BMI) and sex [22]. Thus far, the profile of fecal microbial communities in Chinese IgAN patients remains unclear. Therefore, in the present study, we aimed to provide new clues for the early diagnosis and treatment of IgAN. We comparatively investigated the microbial communities in the feces of Chinese IgAN patients and healthy controls by using 16S ribosomal RNA (rRNA) gene sequencing and evaluated the relationship between the gut microbiota and clinical features of IgAN.

## Methods

### Subjects

A total of 17 hospitalized patients with IgAN in the Hunan Provincial People’s Hospital and 18 age-, sex-, and BMI-matched healthy controls were enrolled in this study. All participants, including patients and control individuals, were Hunan Province natives and Han Chinese. Before they were enrolled, both the patients and healthy controls provided written informed consent. All clinical tests and methods including collection of feces were performed in accordance with relevant guidelines and regulations. This study was approved by the Medical Ethics Committee of the Hunan Provincial People’s Hospital. Dietary information over the past three months was questioned, such as consumption of refined carbohydrates, meats, eggs, dairy products, vegetables, and fruits. Dietary habits were assessed by frequency: frequently (once every ≤3 days), sometimes (once every 4–7 days), occasionally (once every 8–30 days), and none (once for > 1 month).

### Inclusion and exclusion criteria for IgAN

To avoid the effects of drugs and other hospital factors on intestinal microorganisms, we included only newly diagnosed IgAN patients without any prior treatment and collected inpatient samples before starting any medication. Samples were further screened after the diagnosis of IgAN was confirmed by renal biopsy. Thus, these patients did not take medications before sample collection. Renal biopsy specimens were evaluated by the presence of four histological features: mesangial hypercellularity (M), endocapillary hypercellularity (E), segmental glomerulosclerosis (S), and tubular atrophy/interstitial fibrosis (T), as defined in the Oxford-MEST classification. The exclusion criteria were as follows: secondary IgAN, such as lupus nephritis, hepatitis B-associated nephritis, and anaphylactoid purpura nephritis; IgAN patients using hormones, angiotensin-converting-enzyme inhibitors/angiotensin II receptor blockers or immunosuppressants; individuals who took antibiotics, vitamins, probiotics, prebiotics, and laxatives within three months prior to sample collection; individuals with a history of cholecystectomy, colectomy or other intestinal diseases; and individuals with diarrhea, constipation, serious infections and complications.

In addition, age-, sex-, and BMI-matched healthy individuals were recruited as controls. These volunteers came to our hospital for a routine medical examination, which confirmed that they were in healthy condition and not taking medications. As for the family members of the patients, since most of them did not have a medical examination and were experiencing a long-term anxiety about the patients’ suffering, it was unacceptable to select patients’ family members as the control individuals.

### Sample size and power analysis

Sample size and powder analysis were calculated with the help of the G-Power software V3.1.9.4 [23, 24]. The threshold parameter was set by default to type I error of 0.05 and a potency of 80% and effect size of 0.5623, and we calculated that a minimum of 15 samples were required. Effect size was obtained according to preliminary experimental results and demonstrated that the sample sizes we used were adequate to address our research questions.

### Clinical data and biochemical analysis

Personal information, including sex, age, weight, and height, was collected, and BMI was calculated. Fasting peripheral venous blood samples were drawn in the morning, and serum biochemical parameters were analyzed by using automatic biochemical analyzers (Beckman Coulter AU5800, Brea, CA, USA), including blood urea nitrogen (BUN), serum creatinine (sCr), cystatin C (CysC), fasting blood glucose (FBG), total cholesterol (TC), and triglycerides (TG). On the first morning, urine samples were collected and centrifuged; then,urinary albumin (uALB) was quantified by a turbidimetric inhibition immune assay, urine creatinine (uCr) was determined with a sarcosine oxidase assay, C-reactive protein (CRP) was quantified by immunoturbidimetric assay and the estimated glomerular filtration rate (eGFR) was calculated using the Chronic Kidney Disease Epidemiology Collaboration (CKD-EPI) equation [25].

### Fecal sample collection and DNA extraction

Fecal samples were collected using sterile harvesters and frozen in sterile cryotubes at − 80 °C in no more than 30 min. Bacterial DNA was extracted from the fecal samples using the HiPure Stool DNA Kit (Magen, Guangzhou, China) according to the manufacturer’ instructions.

### PCR amplification and amplicon sequencing

The V3-V4 region of the 16S rDNA gene from the fecal DNA was amplified using the barcoded primers 341F (CCTACGGGNGGCWGCAG) and 806R (GGACTACHVGGGTATCTAAT). PCR amplification was carried out in a 50 μL reaction system containing 5 μL of 10× KOD buffer, 5 μL of 2.5 mM dNTPs, 1.5 μL of 5 μM primers, 1 μL of KOD polymerase, and 100 ng of template DNA. PCRs were performed using the following cycle conditions: predenaturation at 95 °C for 2 min, followed by 27 cycles of denaturation at 98 °C for 10 s, annealing at 62 °C for 30 s, extension at 68 °C for 30 s, and a final extension at 68 °C for 10 min. The amplified products were purified using the AxyPrep DNA Gel Extraction Kit (Axygen Biosciences, Union City, CA, USA) and then sequenced on the Illumina HiSeq 2500 platform by Gene Denovo Biotechnology. (Guangzhou, China).

### Data processing and analysis

The raw data was subjected to vigorous quality control, tag splicing, tag filtering, tag dechimerization, and deredundancy processing to obtain effective sequence tags; Uparse software was used to cluster all effective tags of the samples to the same operational taxonomic units (OTUs) based on amplicon sequence similarity ≥97%. The most abundant sequence from each OTU was selected as the representative sequence for species annotations by using the Ribosomal Database Project (RDP) Naive Bayes classifier. After annotation, all OTUs < 10 or proportion < 1% were discarded in the diversity analysis progress. In addition, α and β diversity of microbial communities was analyzed using the standard Quantitative Insights into Microbial Ecology (QIIME) software. For β diversity analysis, the Weighted UniFrac based on phylogenetic distances was used to assess the similarities between a pair of samples. Principal coordinate analysis (PCoA) was performed by multivariate analyses to compare the differences between the microbial community distances of the samples. Functional analyses of microbial communities were performed using the software Tax4Fun. SPSS (ver. 22.0) and R software (ver. 3.1.0) were used for the statistical analysis. Comparisons between continuous variables were performed using the Student *t* test. Wilcoxon rank sum test was used to compare the flora abundance of the two groups. The association between categorical variables was investigated by Chi-square or Fisher exact tests. Spearman correlation analysis was used to evaluate the correlation between fecal microbiota and IgAN clinical parameters, and random forest (RF) model was used to evaluate the disease status based on microbiota profiles (significantly different taxa at genus level from Wilcoxon rank sum test). *P* < 0.05 was considered statistically significant.

### Experimental validation in public database

Gut microbiota data associated with all kidney diseases were downloaded from database of human gut microbiota (*https://gmrepo.humangut.info/home*) and employed as reference data. Up to december 2019, a total of 163 samples associated with kidney diseases were downloaded from the database of human gut microbiota. Only those gut microbiota which were found in ≥ two samples and with median relative abundances ≥0.01% were taken into consideration. The relative abundances of all gut microbes (OTUs) downloaded from the database of human gut microbiota were calculated by R software. All annotated results (OTUs) in our study were compared to the public data.

## Results

### Basic clinical and biochemical characteristics of IgAN patients and healthy controls

The individual demographics, biochemical characteristics, eating habits, and pathological features of the studied subjects are summarized in Table 1. There were no significant differences between the IgAN patients and healthy controls in age, sex, and BMI as well as the BUN, glucose, TC, and TG levels. However, serum CysC and sCr, the urinary albumin-to-creatinine ratio (uACR), and blood CRP were significantly increased, but eGFR was significantly reduced in the IgAN patients when compared to the healthy controls. Moreover, there were no significant differences in dietary habits between the two groups (Table 2).

**Table 1:** Clinical and demographic features of IgAN patients and healthy controls

	Healthy group (*n* = 18)	IgAN group (*n* = 17)	***P***-value
Sex (Female/Male)	4/14	3/14	0.735
Age (years)	49.72 ± 8.39	44.76 ± 10.53	0.132
BMI (kg/m^2^)	23.16 ± 1.39	22.18 ± 2.09	0.108
CysC (mg/L)	0.55 ± 0.08	0.82 ± 0.43	0.011
BUN (mmol/L)	4.88 ± 0.99	5.64 ± 2.29	0.208
Scr (μmol/L)	56.35 ± 8.65	101.43 ± 87.04	0.036
eGFR (ml/min/1.73m^2^)	107.13 ± 12.52	83.16 ± 32.49	0.006
uACR (mg/g)	9.61 ± 3.00	949.49 ± 814.11	0.000
GLU (mmol/L)	5.03 ± 0.54	5.08 ± 0.51	0.764
TC (mmol/L)	4.43 ± 0.71	4.84 ± 0.99	0.160
TG (mmol/L)	1.29 ± 0.23	1.54 ± 0.62	0.119
CRP	0.47 ± 0.29	1.12 ± 1.17	0.030
Frequency of pathologic features (percentages) in 17 biopsies scored according to Oxford Classification			
mesangial hypercellularity (M) M0 = 12% M1 = 88%			
endocapillary hypercellularity (E) E0 = 88% E1 = 12%			
segmental glomerulosclerosis (S) S0 = 71% S1 = 29%			
tubular atrophy/interstitial fibrosis (T) T0 = 76% T1 = 18% T2 = 6%			

Note: Results are expressed as the mean ± SD and ratio. Abbreviations: *SD* standard deviation; *BMI* body mass index; *CysC* Cystatin C; *BUN* blood urea nitrogen; *Cr* creatinine; *eGFR* estimated glomerular filtration rate; *uACR* urinary albumin-to-creatinine ratio; *GLU* glucose; *TC* total cholesterol; *TG* Triglyceride; *CRP* C-reactive protein

**Table 2:** Frequency list of dietary habits in IgAN patients and healthy controls

	Healthy group (F/S/O/N)	IgAN group (F/S/O/N)	***P***-value
Refined carbohydrates	13/3/2/0	12/4/1/0	0.783
Meats and Eggs	10/6/2/0	11/4/2/0	0.811
Dairy products	8/6/2/2	7/5/2/3	0.954
Vegetables and fruits	14/3/1/0	13/2/2/0	0.763
intotal	18	17	

Note: *F* frequently; *S* sometimes; *O* occasionally; *N* none;

### α and β diversity between IgAN patients and healthy controls

**Fig. 1:**
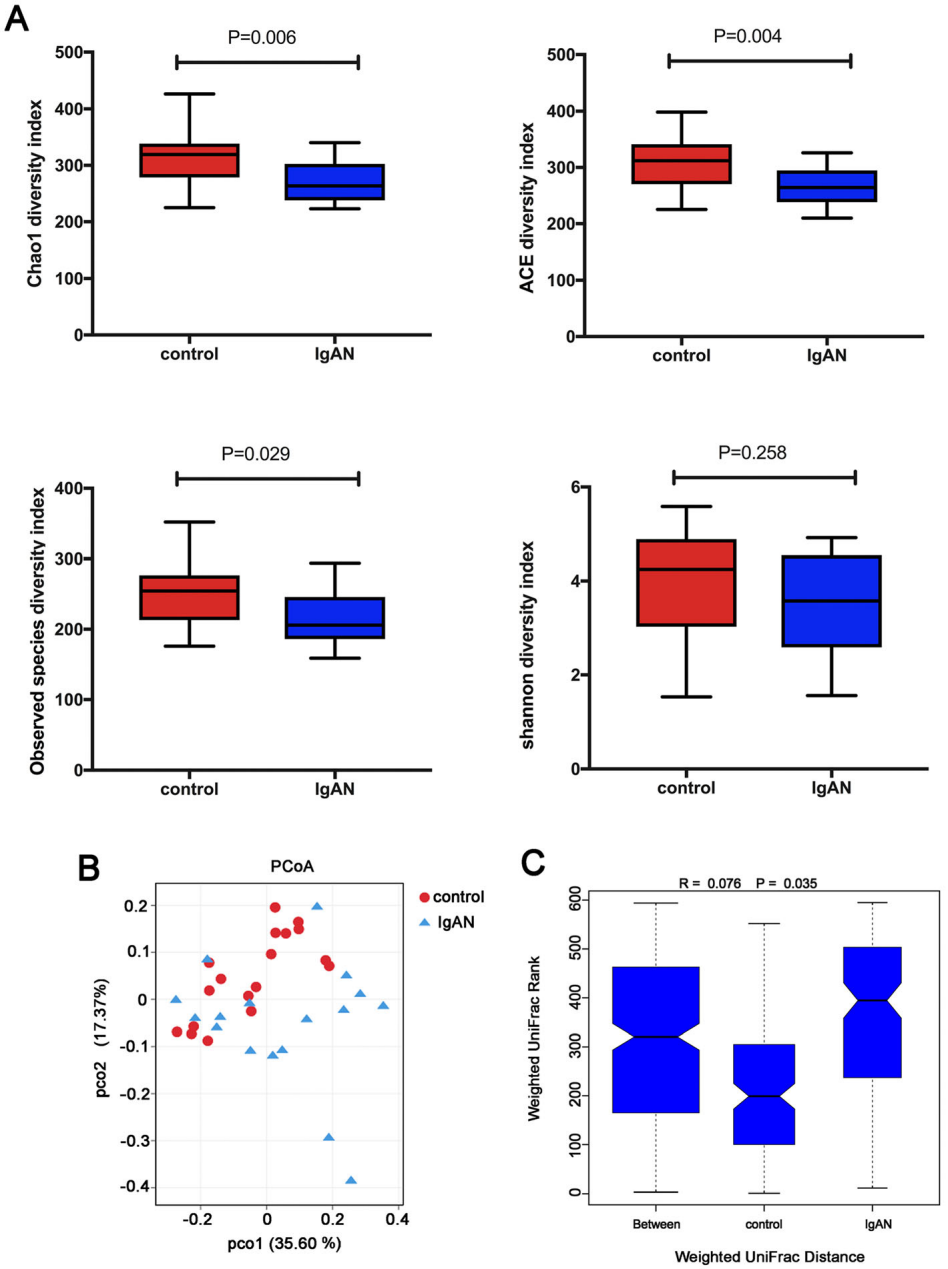
Comparison of diversity indexes between the IgAN patients and healthy controls. (**a**) The differences of α-diversity indexes (Chao1, ACE, Observed species, and Shannon index) between the IgAN patients and healthy controls. (**b**) Principal coordinates analysis (PCoA) with weighted UniFrac distance for bacterial communities between the IgAN patients and healthy controls. (**c**) Analysis of similarities (ANOSIM) based on the weighted UniFrac distance between the IgAN patients and healthy controls

To contrast the microbial community richness and composition between the IgAN patients and healthy controls, we evaluated the α and β diversity of fecal microbiota. With the exception of the Shannon diversity index, other α diversity indexes, including Chao, ACE, and observed species diversity, were significantly decreased in the IgAN patients compared to those in the healthy controls (Fig. 1a), indicating that the microbial community richness of the gut microbiota is significantly lower in the IgAN patients than in the healthy controls. Based on the PCoA plot, we found a separation trend in the β diversity between the IgAN patients and healthy controls (Fig. 1b). Also, a significant difference in β-diversity was observed between the two groups based on the weighted UniFrac clustering (ANOSIM R = 0.076, *P* = 0.035; Fig. 1c), suggesting that the fecal microbial community structure in the IgAN patients is significantly different from that in the healthy controls according to the presence of fecal OTUs.

### Bacterial taxa differences between IgAN patients and healthy controls

**Fig. 2:**
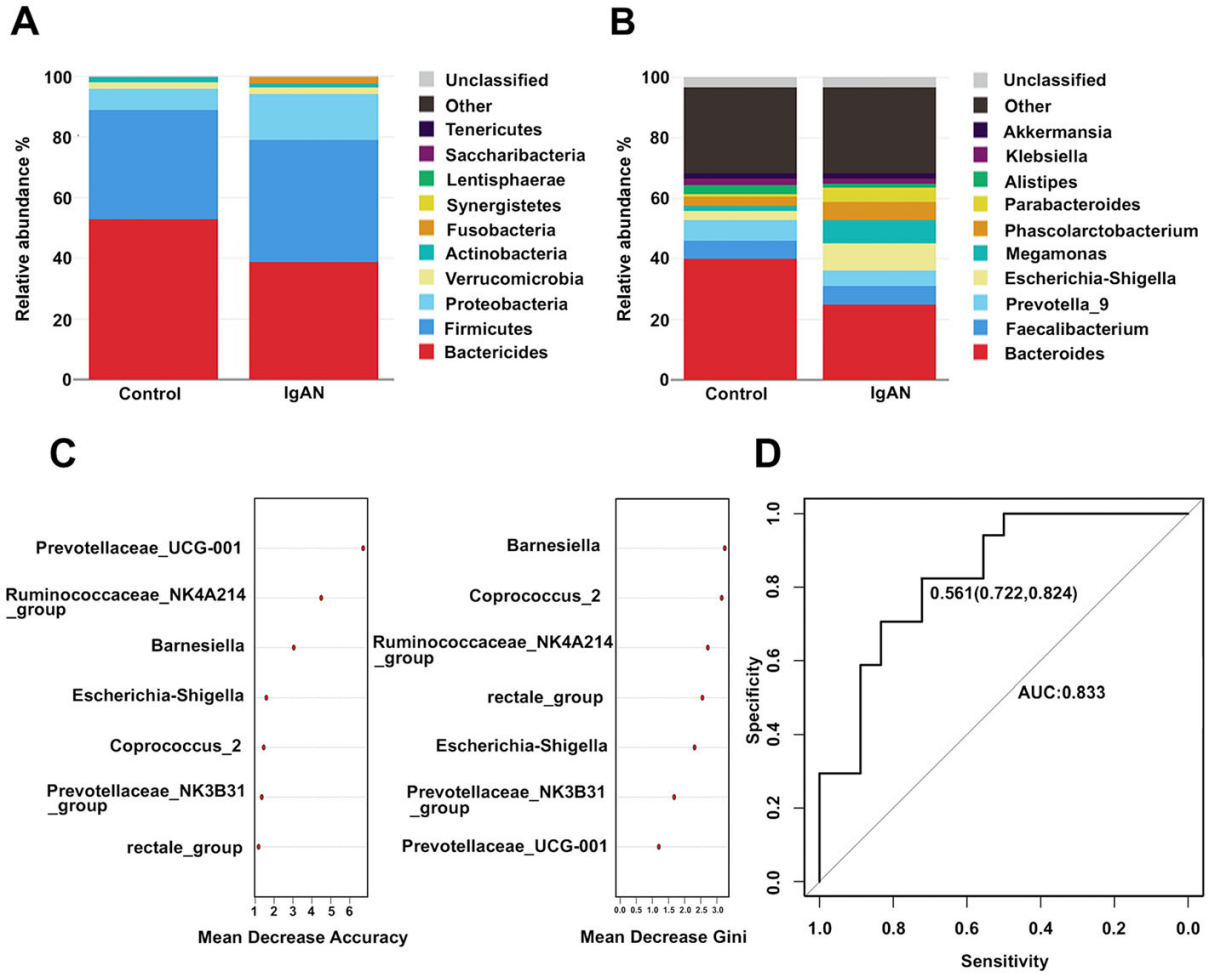
Relative abundance and taxonomic differences of fecal microbiota in the IgAN patients and healthy controls. (**a**) Microbiome composition in the two groups at the phylum level. (**b**) Microbiome composition in the two groups at the genus level. The figure shows the top 10 species in each group based on their relative abundances. (**c**) The predictive model based on the relative abundances at genus level by using a RF model. The importance of each genus in the predictive model was evaluated by the mean decreasing accuracy and the Gini coefficient. (**d**) ROC curve analysis generated by the RF using 7 genera in the fecal microbiota

Both IgAN patients and healthy controls had typical microbiome structures. Most bacteria fell within the phyla *Bacteroidetes, Firmicutes, Proteobacteria,* and *Verrucomicrobia* (Fig. 2a). There was no statistically significant difference in the microbiome structure between the two groups, although the IgAN patients showed an increased abundance of *Firmicutes* and a decreased abundance of *Bacteroidetes* and *Proteobacteria*. Note that compared to the healthy controls, the IgAN patients showed an increased abundance of *Fusobacteria* but a decreased abundance of *Synergistes* (*P* < 0.05). Within *Proteobacteria*, the *Enterobacteriaceae* abundance was significantly higher in the IgAN patients than in the healthy controls at the family level (*P* < 0.05). For the *Synergistetes* family, a significant decrease was found in the *Synergistaceae* abundance in the IgAN group compared to that in the healthy group (*P* < 0.05) (Table 3).

**Table 3:** Relative abundance of fecal microbiota in IgAN patients and healthy controls

	Healthy group	IgAN group	***P***-value
Phylum level			
*p- Fusobacteria*	0.2269	2.2622	0.031
*p- Synergistetes*	0.0297	0.0097	0.026
Family level			
*f- Enterobacteriaceae*	5.9760	12.4897	0.031
*f- Synergistaceae*	0.0297	0.0097	0.026
Genus level			
*g- Escherichia-Shigella*	3.0358	8.8629	0.048
*g- rectale_group*	2.5503	0.9346	0.018
*g- Barnesiella*	0.5212	0.2121	0.021
*g- Ruminococcaceae_NK4A214_group*	0.3045	0.0806	0.024
*g- Prevotellaceae_NK3B31_group*	0.1767	0.1188	0.011
*g- Prevotellaceae_UCG-001*	0.2161	7.059e-05	0.003
*g- Coprococcus_2*	0.1134	0.05403	0.007
*g- Hungatella*	0.0062	0.1444	0.026
*g- Rikenellaceae_RC9_gut_group*	0.1037	0	0.002
*g- Lachnospiraceae_FCS020_group*	0.0414	0.0187	0.014
*g- Eggerthella*	0.0156	0.0417	0.001
*g- Pyramidobacter*	0.0293	0.0089	0.004

Note: Only *P*-values < 0.05 are shown

At the genus level, *Bacteroides, Faecalibacterium, Prevotella-9, Escherichia-Shigella,* and *Megamonas* were the most abundant genera in both groups. Some of these genera showed a significant difference between these two groups (Fig. 2b). The abundances of the genera *Escherichia-Shigella*, *Hungatella,* and *Eggerthella* were higher in the IgAN group than in the healthy group, whereas the abundances of the genera *rectale_group, Barnesiella, Ruminococcaceae_NK4A214_group, Prevotellaceae_NK3B31_group, Prevotellaceae_UCG-001 Coprococcus_2, Lachnospiraceae_FCS020_group,* and *Pyramidobacter* were higher in the healthy group than in the IgAN group (*P* < 0.05) (Table 3). These data suggest that the taxonomic abundances of microbial communities are different in the IgAN patients and the healthy controls.

### Random forest (RF) predictive modeling

To evaluate the disease status of the IgAN patients based on an ensemble of decision trees, we used RF to build a predictive model according to the fecal microbiota profiles with the significantly different taxa abundances at the genus level from Wilcoxon rank-sum test as the input. Among the fecal microbiota, 7 genera predicted IgAN with a sensitivity of 82.4% and a specificity of 72.2% (AUC = 0.833, 95% CI: 0.699–0.967) using the RF model (Fig. 2c and d), suggesting that certain genera of fecal microbiota are predictive of the IgAN disease state.

### Association between fecal microbiota and IgAN clinical characteristics

**Fig. 3:**
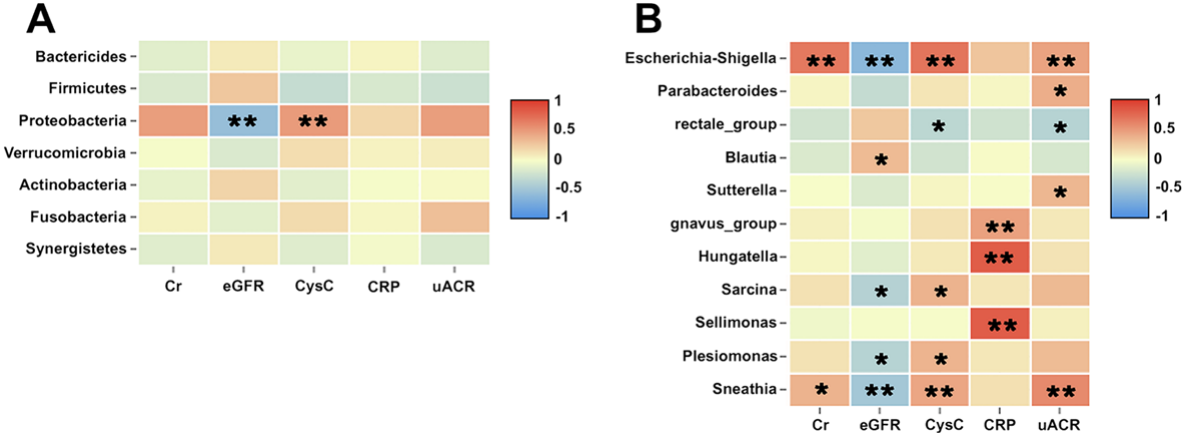
Heatmaps showing correlations between fecal microbiota and IgAN clinical parameters. (**a**) Correlations between microbiota phyla and IgAN clinical characteristics. (**b**) Correlations between microbiota genera and IgAN clinical characteristics. The intensity of the color indicates the r value (correlation). The red color represent positive score and the blue color represent negative score. *P < 0.05 and **P < 0.01

We evaluated the correlation between fecal microbiota (at the phylum level and genus level) and IgAN clinical parameters (Fig. 3) and found that the phylum *Proteobacteria* and the genera *Escherichia-Shigella, Sneathia,* and *Plesiomonas* were negatively associated with the eGFR, while the genus *Blautia* was positively associated with the eGFR. Moreover, the genera *Escherichia-Shigella, Sneathia,* and *Parabacteroides* were positively associated with the uACR, but the genus *rectale_group* was negatively associated with the uACR. Additionally, the genera *Hungatella, Sellimonas,* and *gnavus_group* were positively associated with CRP. These findings suggest a strong correlation of altered fecal microbiota with the clinical features of the IgAN patients.

### Functional analysis

**Fig. 4:**
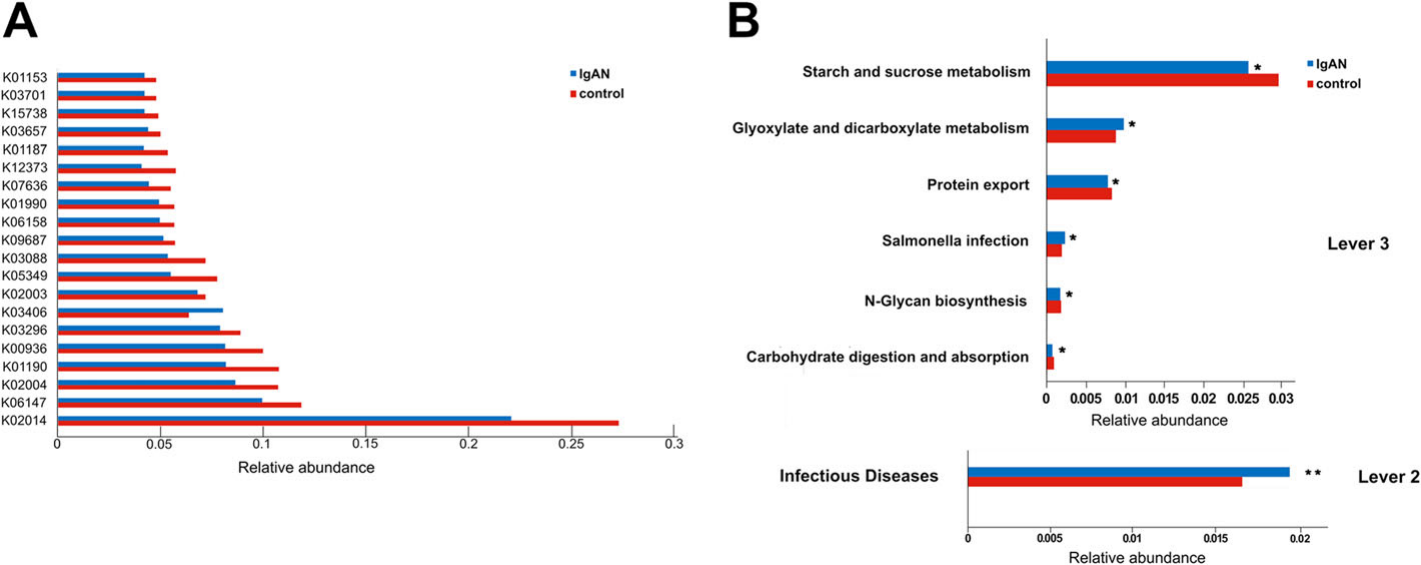
Predicted microbial functions using Tax4fun. (**a**) The top 20 KOs with significantly different abundances between the IgAN patients and healthy controls. (**b**) Significant KEGG pathways at level 2 and 3 between the IgAN patients and healthy controls. *P < 0.05 and **P < 0.01

Tax4Fun [26] based on the SILVA rRNA database as a reference was used to predict the relative abundances of functional categories from databases such as the Kyoto Encyclopedia of Genes and Genomes (KEGG) ortholog (KO) database. Our results show differential KOs identified between the IgAN and healthy groups (*P* < 0.05; Fig. 4a). At levels 2 and 3 of the KEGG pathways, the microbial gene functions, including those pathways involved in infectious diseases, glyoxylate and dicarboxylate metabolism, and *Salmonella* infection, were higher in the fecal microbiome of the IgAN group, whereas the microbial gene functions related to starch and sucrose metabolism, N-glycan biosynthesis, carbohydrate digestion and absorption, and protein export were higher in the fecal microbiome of the healthy group (*P* < 0.05; Fig. 4b), indicating the differential function of the fecal microbiota between diseased and healthy individuals.

### In comparison to public database

Of all tested genera, 101 microbial genera including *Escherichia-Shigella*, *Bacteroides*, *Klebsiella*, *Fusobacterium* were found in both our study and the pubic database, and 79 microbial genera unique to our study were also found (Supplementary materials 1). In addition, The significantly differential genera between disease and healthy groups from the database of human gut microbiota and our sequencing data were compared. The abundance of the genus *Escherichia-Shigella* was higher in the disease group, and the abundances of the genera *Barnesiella* and *Pyramidobacter* were lower in the disease group. Unfortunately, the genus *Eggerthella* was found decreased in the database of human gut microbiota for kidney diseases, but increased in our study. The genera *Hungatella*, *rectale_group*, *Coprococcus_2* and *Lachnospiraceae_FCS020_group* that were significantly different in our study were not found in the database (no relative abundance results were annotated). These results suggest that all patients with kidney diseases may share certain core microorganisms regardless of the impact of the patients’ survival environment and etiology, while some bacterial taxa may be related to the pathological characteristics of IgA nephropathy.

## Discussion

Previous studies have reported that patients with celiac disease and inflammatory bowel disease are more likely to develop IgAN [27–29]. The immune system responds vigorously to microbial infection while permitting life-long colonization by the microbiome. The microbiome could mediate stable colonization of the gut through coopting IgA responses [30]. Differences in the gut microbial composition and diversity have been observed in Italian Caucasians with IgAN and are closely associated with clinical phenotype [21]. Here, we determined for the first time the fecal microbial composition in Chinese patients with primary IgAN. Our results demonstrated that the community richness of fecal microbiota in the IgAN patients was significantly lower than that in the healthy controls, in agreement with another observation [21]. Note that we confirmed a significant difference in the β-diversity indexes between the diseased and healthy subjects, which has not been previously reported. These findings indicate that fecal microbiota dysbiosis occurs in Chinese patients with primary IgAN. Taxonomic differences in fecal microbiota between Italian patients and Chinese patients may be due to distinct cultural backgrounds, geographical locations, and eating habits. It has been reported that there are significant differences in the intestinal bacterial diversity among populations of different cultures and geographical regions [31], and dietary factors largely affect microbial composition and function [32]. Therefore, it is important to explore the etiology and therapeutic approach of intestinal flora in the Chinese population.

We found that at the phylum level, *Bacteroidetes*, *Firmicutes*, and *Proteobacteria* were the dominant bacteria in all tested samples. This observation is in agreement with a previous report that patients with nonprogressive or advanced IgAN had a low abundance of *Bacteroidetes* but a high abundance of *Firmicutes* [21]. We also found that the abundance of *Synergistetes*, mainly related to the decreased abundance of the genus *Pyramidobacter*, was significantly decreased in IgAN patients. As ruminal microbes, *Pyramidobacter* is believed to participate in the synthesis of thiamine [33, 34], which is important for the maintenance of gut-associated lymphoid tissues [35, 36]. Therefore, it is reasonable to speculate that *Pyramidobacter* may provide some health benefits to patients with IgAN. More importantly, IgAN patients had an increased abundance of *Fusobacteria, Proteobacteria, Enterobacteriaceae,* and the specific genus *Escherichia-Shigella*, most of which are opportunistic pathogens. *Fusobacteria* are gram-negative obligate anaerobic nonspore-forming bacilli that represent a small proportion of the microbial community, and clinically invasive *Fusobacterial* infections are rare [37]. Nevertheless, recent studies have shown that *Fusobacteria* are associated with Crohn’s disease, ulcerative colitis, and colorectal cancer [38]. Moreover, the enrichment of *Proteobacteria* is considered a potential microbial diagnostic signature of dysbiosis and increases the risk of host diseases [39]. As the most common commensal flora in the host-mediated inflammatory response, the excessive proliferation of *Enterobacteriaceae* driven by inflammation is closely related to the development of various human diseases [40]. For example, hyperproliferative *Enterobacteriaceae* is present in the feces of ESRD patients [41], and bacterial DNA from the genera *Escherichia* and *Enterobacter* is also detectable in the blood of ESRD patients [42]. We also found that some genera that produce short-chain fatty acids (SCFAs), including *Ruminococcaceae_NK4A214_group, Coprococcus_2,* and *Lachnospiraceae_FCS020_group,* became less in the IgAN patients. SCFAs are generally considered to have a number of important roles in maintaining health, such as acting as a special nutrient and energy source for the intestinal epithelium, protecting the intestinal mucosal barrier, reducing inflammation, and enhancing the motility of the gastrointestinal tract [43]. *Barnesiella* is one of the most abundant genera detected in the mouse intestine, has anti-inflammatory protective effects in animal models [44, 45], and was found to be significantly reduced in the IgAN patients in this study. Recent studies observed that the genus *Barnesiella* was the most abundant novel classification in the healthy human gut microbiome [46] and correlated with a healthy state of subjects [47]. The major end products derived from *Barnesiella* are butyric and isobutyric acids [48], which are responsible for the beneficial effects of the bacteria. Furthermore, members of the *Prevotellaceae* possess phosphotransbutyrylase and butyrate kinase, which are key enzymes in the butyrate synthesis pathway [49]. *Prevotellaceae_NK3B31_group* and *Prevotellaceae_UCG-001* of the *Prevotellaceae* family were also found to be significantly decreased in the IgAN patients. The reduced abundance of *Prevotellaceae* has also been reported in the stool of ESRD patients [49] and uremia animals [50]. It is well known that intestinal epithelial cells are the first line of cellular defense against pathogen invasion, and that butyrate serves as the primary energy source for intestinal epithelial cells. Butyrate not only regulates stem cell turnover in the intestinal epithelial crypt, but also enhances the bactericidal function of macrophages by shaping their transcription and metabolism [51]. Collectively, the presence of IgAN is related to the abundant change in some bacterial taxa. Our RF models successfully predicted IgAN using several differential several genera with a high sensitivity and specificity. Spearman correlation analysis confirmed that some opportunistic pathogens, such as *Escherichia-Shigella* and *Sneathia,* were positively correlated with the uACR but negatively correlated with the eGFR, both of which are classical renal damage markers. We also found that the genus *rectale_group* was negatively correlated with the uACR and that the genus *Blautia* was positively associated with the eGFR. Additionally, by comparing with the database, we found that 101 genera in our study were consistent with the public database, which indirectly validated the accuracy of our results. And some new potential kidney disease-related microorganisms were annotated in our study, but not found in the public database, which may enrich the public data of kidney-related gut microorganisms. At the same time, we also found that the genera *Escherichia-Shigella*, *Barnesiella*, and *Pyramidobacter* showed the same variation characters in known public database and our study, suggesting their potential role as biomarkers for kidney disease. In contract to public database, we found that the abundance of *Eggerthella* was significantly increased in IgAN patients. The implication of *Eggerthella* in inflammatory diseases have been reported in numerous studies [52]. For example, the increased abundance of *Eggerthella* was associated with type II diabetes [53], rheumatoid arthritis [54] and Crohn’s disease [55], which maybe support the phenomena of our study. Taken together, these results indicate that changes in the composition of specific bacteria may be helpful for the early diagnosis and prediction of IgAN.

Studies have shown that individuals with a low bacterial richness are characterized by a more inflammatory phenotype [56]. The association between increased *Enterobacter* and decreased renal function has been confirmed in many studies [57, 58]. The mechanisms behind these phenomena may be attributable to the inflammatory immune response and intestinal susceptibility of IgAN. For instance, the mucosal infection of IgAN patients triggers hematuria and damages the intestine, resulting in acute diarrhea [10]. IgAN patients usually show increased intestinal permeability [59], which facilitates the absorption and circulation of bacterial LPS derived from the *Escherichia* genus [60]. Animal studies [61] have demonstrated that the expansion of *Proteobacteria* (specifically *Escherichia-Shigella*) and enrichment of genes involved in LPS biosynthesis are parallel to the increased levels of LPS in the feces and circulation. LPS and transmembrane signal transduction holds the key to the inflammatory effects of cells. By analyzing mesangial cell proteomics and glomerular transcriptomics, Liu et al. [62] found that most identified pathways induced in mesangial cells by galactose-deficient IgA are involved in inflammation. A link between LPS exposure and defective IgA galactosylation has been confirmed by a study showing that bacterial LPS activates TLR4 in cultured peripheral B lymphocytes to induce Cosmc expression critical for the activity of the enzyme galactosyltransferase [63]. TLR4, the receptor for LPS from *Escherichia coli*, has been reported with increased mRNA expression in peripheral lymphomononuclear cells of patients with IgAN and in children with IgA vasculitis; in addition, TLR4 is correlated with the signs of innate immunity activation and proteinuria [64, 65]. Pathway analyses based on KEGG analysis in our study demonstrated that the gut microbiota in patients with IgAN is enriched in multiple pathways associated with infectious diseases. This finding further supports the concept that systemic immune activation and inflammation triggered by gut microbiota-associated LPS signaling play an important role in the development and progression of IgAN. In addition, it is generally believed that the availability of carbohydrates and nitrogen is the most important determinant of microbial metabolism in the colon. In the absence of carbohydrates, α-amino nitrogen can produce potentially toxic end products mainly through fermentation. It has been reported that the α-amino nitrogen fermentation pattern is the key player in gut ecosystem disorders in CKD patients [66], which may contribute to IgAN.

It should be noted that our study has certain limitations. First, this is a cross-sectional study. To reduce the effects of individual differences, a longitudinal study focusing on microbial differences at the different stages of IgAN is needed. Second, our sample size is relatively small; therefore, a large-scale study involving different populations is needed to confirm our results. Third, although Tax4Fun provides functional information about gut microbiota, its limitation is obvious compared with the information provided by shotgun metagenomics analysis. Nevertheless, we generated solid data supporting the role of altered gut microbiota in the pathogenesis of Chinese IgAN patients in this well-matched (i.e., age, BMI, consistency of medication) cohort with the use of next-generation sequencing of specific genomic regions of the microbiome.

## Conclusions

Overall, we collected evidence for gut microbiota dysbiosis in IgAN patients from the Hunan region of China. Our finding regarding the relation of some fecal microbiota to IgAN clinical characteristics will surely help enhance our understanding of the pathogenesis of IgAN and provide potential novel therapeutic options for patients with IgAN by targeting gut microbiota.

## Supplementary information

**Additional file 1: Supplementary materials 1.** Comparison of all genera between our result and public database.

## Data Availability

The datasets used and analyzed in this study are available from the first author and corresponding author on reasonable request.

## Abbreviations

BMI: Body mass index
eGFR: Estimated glomerular filtration rate
ESRD: End-stage renal disease
IgAN: Immunoglobulin A nephropathy
LPS: lipopolysaccharide
RF: Random forest
uACR: Urinary albumin-to-creatinine ratio
